# Using Polygenic Profiles to Predict Variation in Language and Psychosocial Outcomes in Early and Middle Childhood

**DOI:** 10.1044/2019_JSLHR-L-19-0001

**Published:** 2019-08-19

**Authors:** Dianne F. Newbury, Jenny L. Gibson, Gina Conti-Ramsden, Andrew Pickles, Kevin Durkin, Umar Toseeb

**Affiliations:** a Department of Biological and Medical Sciences, Headington Campus, Oxford Brookes University, United Kingdom; b Faculty of Education, University of Cambridge, United Kingdom; c School of Health Sciences, The University of Manchester, United Kingdom; d Department of Biostatistics, Institute of Psychiatry, King's College London, United Kingdom; e School of Psychological Sciences, University of Strathclyde, United Kingdom; f Department of Education, Derwent College, University of York, United Kingdom

## Abstract

**Purpose:**

Children with poor language tend to have worse psychosocial outcomes compared to their typically developing peers. The most common explanations for such adversities focus on developmental psychological processes whereby poor language triggers psychosocial difficulties. Here, we investigate the possibility of shared biological effects by considering whether the same genetic variants, which are thought to influence language development, are also predictors of elevated psychosocial difficulties during childhood.

**Method:**

Using data from the U.K.-based Avon Longitudinal Study of Parents and Children, we created a number of multi–single-nucleotide polymorphism polygenic profile scores, based on language and reading candidate genes (*ATP2C2, CMIP, CNTNAP2, DCDC2, FOXP2,* and *KIAA0319,* 1,229 single-nucleotide polymorphisms) in a sample of 5,435 children.

**Results:**

A polygenic profile score for expressive language (8 years) that was created in a discovery sample (*n* = 2,718) predicted not only expressive language (8 years) but also peer problems (11 years) in a replication sample (*n* = 2,717).

**Conclusions:**

These findings provide a proof of concept for the use of such a polygenic approach in child language research when larger data sets become available. Our indicative findings suggest consideration should be given to concurrent intervention targeting both linguistic and psychosocial development as early language interventions may not stave off later psychosocial difficulties in children.

There is sound evidence for the heritable nature of language ability in children (Stromswold, 2001). The evidence concerning specific gene contributions is weaker: Typically, single gene analyses account for only a very small proportion of trait variance or disorder (Simpson et al., 2015). Relationships vary between genetic variants within individuals, across populations, and across genes meaning that the identification of a single variant is far from straightforward. This often means that the effects of variants do not replicate across studies or show differences in effect direction between cohorts. Within the current literature, it is hard to ascertain whether such variants represent false positives or heterogeneous effects (Nudel, Ceroni, Simpson, & Newbury, 2015). This state of affairs has led researchers interested in genetic effects to aggregate indicative findings concerning the relationships between specific alleles and particular traits into polygenic profile scores. The advantage of polygenic profiles is that they combine information from multiple variants and across genetic loci, enabling more comprehensive and powerful tests of the hypothesized “genetic burden” associated with a condition (Levine et al., 2014; Mancuso et al., 2018; Wray et al., 2014).

The formation of polygenic profiles begins with a direct genetic association model within a large “discovery data set.” This involves the investigation of correlations between individual genetic variants (usually single-nucleotide polymorphisms or SNPs) and a behavioral outcome of interest. All SNPs that show association above a certain threshold (in terms of *p* value or effect size) are then collapsed into a single weighted composite measure of genetic effects across multiple variants. Typically, scores are calculated across a range of thresholds in the discovery sample to allow the identification of the cutoff that confers maximal predictive power within the discovery sample set. Scores allow for the presence of false-positive associations, and data sets can be pruned for relationships between genetic variants (clumping). Once formulated, the polygenic profile score can be used within a linear regression model to predict outcomes in a second independent “replication cohort.” This may be performed for any measure that is expected to show shared genetic effects with the behavioral measure of interest. The prediction accuracy is usually stated as an *R* ^2^ measure, which represents the proportion of variance explained. Polygenic methods have proven particularly successful in disorders in which combinations of common variants predispose individuals to increased risk. Polygenic profiling has been used to indicate significant genetic overlaps between neuropsychiatric disorders (Ritter et al., 2017) as well as between cognitive ability and educational outcomes (Krapohl et al., 2018).

Such disorders are typically modeled under a “common disease-common variant” model. While genetic sequencing studies indicate that rare variants are also likely to play a role in neuropsychiatric disorders and traits, these will not be captured by polygenic profiling and are unlikely to be important in individual differences in language ability. In most instances, the overall predictive power of polygenic profiles remains low in terms of individual risk. Nonetheless, such profiles allow the identification of individuals at a particularly high risk and can provide accurate indictors of the underlying risk interactions providing important information for risk modeling.

Given the lack of large-scale genome-wide association studies (GWAS) for language-related measures, we selected robust candidate genes from the literature on genes on language and reading abilities and/or disorders to construct targeted scores in this study. Studies of targeted genes have indicated that some genes may have impacts across traits and disorders (Newbury et al., 2011; Scerri et al., 2011). Variants within the *KIAA0319* and *CNTNAP2* genes have been associated with both dyslexia and language impairment as well as reading and language measures in the general population (Newbury et al., 2011; Rice, Smith, & Gayán, 2009). In contrast, variants in *DCDC2* seem to be of particular relevance to reading disability (Scerri et al., 2011) and, those in *ATP2C2*, to language impairment (Newbury et al., 2009). *FOXP2* appears to play a very specific role but may only be relevant in the presence of coding mutations with high effect sizes (Morgan, Fisher, Scheffer, & Hildebrand, 2016). It has been suggested that variants within the *CMIP* gene may have alternative effects within different populations; the “risk” allele of rs12927866 has been associated with lower performance in tests of nonword repetition in individuals with language impairment but with higher performance in the general population (Newbury et al., 2009). Such studies suggest that the role of risk variants may be modulated by the environment or genetic background of an individual and mirror findings of differential susceptibility in the psychiatry literature (Belsky, 1997).

In this study, we applied a polygenic approach in which we consider the effects of robust language and reading candidate genes within a single model. Specifically, we were interested in whether polygenic profiles, based on language and reading candidate genes (*ATP2C2, CMIP, CNTNAP2, DCDC2, FOXP2,* and *KIAA0319*), can be used to provide consistent predictors of language outcomes in early and middle childhood. This was assessed by the generation of polygenic profiles in a discovery cohort and the assessment of their correlation with the same outcomes in an independent replication cohort.

As well as predictors of outcomes, polygenic profiles can also be used to estimate the level of genetic overlap between contributing factors. Since genetic factors have previously been demonstrated to overlap between neurodevelopmental domains and across disorders (Anttila et al., 2018), we sought to investigate the possibility that polygenic profiles for language ability also contribute to the general development process. There is considerable support for this at the behavioral level where individuals with poor language, specifically those with developmental language disorder (DLD), tend to have worse psychosocial outcomes compared to those without DLD (Beitchman et al., 2001; Botting, Durkin, Toseeb, Pickles, & Conti-Ramsden, 2016; Botting, Toseeb, Pickles, Durkin, & Conti-Ramsden, 2016; Durkin, Toseeb, Botting, Pickles, & Conti-Ramsden, 2017; Yew & O'Kearney, 2015a, 2015b).

Such adverse outcomes are not inevitable. While having DLD is certainly associated with risk of poorer psychosocial outcomes compared to unaffected individuals, these are not found invariably and the strength of any relationship may vary across different aspects of psychosocial functioning (Conti-Ramsden et al., 2018; Mok, Pickles, Durkin, & Conti-Ramsden, 2014; Pickles, Durkin, Mok, Toseeb, & Conti-Ramsden, 2016; Toseeb, Pickles, Durkin, Botting, & Conti-Ramsden, 2017). Investigating the etiology of these differences in psychosocial outcomes in individuals with DLD speaks importantly to the debate about whether these problems have a common genetic origin or are linked in a developmental sequence. For example, in the latter explanation, a biological driven language disorder could make social interaction difficult, and hence, psychosocial difficulties (emotional instability, peer relationship problems, conduct disorder, hyperactivity, lack of prosociality) could follow developmentally (Durkin & Conti-Ramsden, 2007; Redmond & Rice, 1998).

While researchers have begun to address the complex task of uncovering genetic factors associated with language ability, scant attention has been paid to the question of whether the same genetic variants predict other characteristics in children with language disorders. In this study, we examined a model of shared genetic effects by considering whether polygenic profiles, based on language and reading candidate genes (*ATP2C2, CMIP, CNTNAP2, DCDC2, FOXP2,* and *KIAA0319*), can be used to provide consistent predictors of psychosocial outcomes in middle childhood.

## Method

### Ethical Approval

Ethical approval for the study was obtained from the Avon Longitudinal Study of Parents and Children (ALSPAC) Ethics and Law Committee and the local research ethics committees. Ethical approval for the secondary analysis of existing ALSPAC data was obtained from the University of York Education Ethics Committee (Reference: 18/5).

### Study Sample

Data from the ALSPAC sample were used in this study. All pregnant women in the old administrative region of Avon, whose estimated delivery was between April 1991 and December 1992, were eligible to participate. The ALSPAC enrolled a sample consisted of 14,775 live-born children from 15,247 pregnancies. This resulted in a total number of 15,458 children (including multiple births). Parents and children provided biological samples and questionnaire data and took part in direct assessments. Full details of the cohort are reported elsewhere (Boyd et al., 2013; Fraser et al., 2013). Data used in this article were mother derived reports on the child and direct assessments of the child by the research team at the following time points: the prenatal period (8 and 32 weeks of gestation) and when the child was aged 15 months, 18 months, 24 months, 8 years, and 11 years. Please note that the study website contains details of all the data that are available through a fully searchable data dictionary and variable search tool (http://www.bristol.ac.uk/alspac/researchers/our-data/).

An initial sample of 15,445 participants was provided by the ALSPAC study team. For this study, the following exclusionary criteria were applied: children who did not have phenotypic data available from speech and language sessions at 8 years old (*n* = 8,062), children who scored below 60 or with incomplete data on performance IQ at 8 years old (*n* = 56), children who were born second in a multiple birth (twins or triplets; *n* = 100), unable to determine DLD status due to missing data or removed from DLD sample due to autism spectrum disorder (ASD) and/or hearing loss (*n* = 475), removed from no-DLD sample due to ASD and/or hearing loss (*n* = 391), missing phenotypic data (*n* = 500), and non-White ethnicity (*n* = 426). This resulted in a study sample of 5,435 (50% male), who were included in the genetic analyses.

### Measures

#### Language Measures

Eight measures of language development were collected. For the parent report measures at 15–24 months, a modified version of the MacArthur–Bates Communicative Development Inventories (Fenson et al., 1993) was used. The MacArthur–Bates Communicative Development Inventories has been shown to have good validity at a population level (Dale, Bates, Reznick, & Morisset, 1989; Feldman et al., 2005).

*Vocabulary at 15 months old.* When the child was 15 months old, parents were given a list of 134 words/phrases and asked whether their child “understands but doesn't say” (1), “understands and says” (2), or “neither” (0). Words/phrases were age appropriate and included words such as “bed,” “nose,” and “hot.” Responses were summed to create a score ranging from 0 to 268. Higher scores indicate better vocabulary.

*Vocabulary at 24 months old.* When the child was 24 months old, a similar measure was used. Parents were given a list of 123 words/phrases and asked whether their child “understands” (1), “says” (2), or “neither” (0). Words were age appropriate and included “hello,” “dinner,” and “chicken.” Responses were summed to create a score ranging from 0 to 246. Higher scores indicate higher vocabulary.

*Receptive language at 15 months old*. Parents were shown a list of 12 phrases and asked whether their child understands. Samples phrases include “Are you sleepy?” and “Don't touch.” Responses were coded on a binary scale (0 = *no*, 1 = *yes*) and then summed to create a score ranging from 0 to 12. Higher scores indicate better receptive language.

*Grammar at 24 months old.* Parents were given four examples of grammar rules and asked whether their child has begun using these rules in their spoken language. Parents were asked about grammar rules such as adding “-ing” to the end of words and adding “-s” to signify plural. Responses were coded as “not yet” (0), “sometimes” (1), or “often” (2) and then summed to create a score ranging from 0 to 8. Higher scores indicate better grammar.

*Receptive language at 8 years old.* The Weschler Objective Language Dimensions (Rust, 1996) was used to measure receptive language. Only one of the two subsets was used in our study. The child was shown a picture and listened to a paragraph about the picture. The child then answered questions about what they had heard. The child was asked 16 questions. Responses were coded on a binary scale (0 = *incorrect*, 1 = *correct*), yielding a summed score of between 0 and 16. Higher scores indicate better receptive language.

*Expressive language at 8 years old*. The Weschler Objective Language Dimensions (Rust, 1996) was used to measure expressive language. Only one of the two subsets was used. The child was shown 10 pictures and asked to name them. Responses were coded on a binary scale (0 = *incorrect*, 1 = *correct*) and then summed to create a score ranging from 0 to 10. Higher scores indicate better expressive language.

*Nonword repetition at 8 years old.* An adapted version of the Nonword Repetition Test (Gathercole, Willis, Baddeley, & Emslie, 1994) was used. The child was asked to listen and repeat out loud four each of three-, four-, and five-syllable nonwords. Responses were coded on a binary scale (0 = *incorrect*, 1 = *correct*) and then summed to create a score ranging from 0 to 12. Higher scores indicate better nonword memory.

*Pragmatic language at 9 years old.* The parent report Children's Communication Checklist (Bishop, 1998) was used to measure pragmatic language when the child was 9 years old. A sum score was created using the sum of the inappropriate initiation, coherence, stereotyped conversation, use of conversational context, and conversational rapport subscales (ranging from 86 to 162). Higher scores indicate better pragmatic language.

#### Psychosocial Measures

The parent report Strengths and Difficulties Questionnaire (SDQ; Goodman, 1997) was used to measure psychosocial outcomes at the age of 11 years. Sum scores were generated for each of the five subscales (Emotional Problems, Peer Problems, Conduct Problems, Hyperactivity, and Prosociality). The scores for each subscale ranged from 0 to 10, with higher scores indicating more difficulties for the problem subscales and higher prosociality.

### DLD Status

Language measures provide an indication of individual differences in language ability but do not always reflect language difficulties, which can be difficult to capture through measures in single domains. We therefore derived a measure of DLD using a previously reported framework (Scerri et al., 2011). Children were categorized as having DLD if they met at least two of the following four criteria: (a) pragmatic language > 1 *SD* below standardized mean, (b) nonword repetition > 1 *SD* below the standardized mean, (c) receptive language > 1 *SD* below the standardized mean, or (d) positive response to “child has ever had speech/language therapy.” In line with recommendations regarding DLD diagnosis, children were excluded if they had a differentiating biomedical condition such as ASD or hearing problems (Bishop, Snowling, Thompson, Greenhalgh, & CATALISE-2 Consortium, 2017). ASD was defined as mothers responding positively that their child had autism, Asperger's, or ASD at the age of 9 years. At the age of 7 years, children underwent a hearing test. Hearing problems were defined as bilateral hearing impairment, left unilateral hearing impairment, or right unilateral hearing impairment. Children with ASD or hearing loss were also excluded from the comparison sample. There were 346 children with DLD, which yielded a prevalence estimate of approximately 6%, which is consistent with prevalence data reported elsewhere (Norbury et al., 2016; Tomblin et al., 1997).

Our approach to identifying children with DLD, which involved selecting children at the tail end of the normal distribution on a number of language measures, allowed us to investigate DLD at a population level. Studies of clinical population may suffer from referral bias and may only represent children at the extreme end of the disorder. The sample of children with DLD that we identified will no doubt be a combination of those with a clinical diagnosis and those who are undiagnosed. Similar approaches have been previously used in other population-wide or community samples (see Forrest, Gibson, Halligan, & St Clair, 2018; Norbury et al., 2016; Reilly et al., 2010).

Descriptive statistics were used to generate profiles of the children with and without DLD. Psycholinguistic and psychosocial characteristics of these two groups, as shown in Table A1 in the Appendix, support the use of such a community-based approach. As a group, children with DLD had poorer early language ability (receptive language: 15 months, vocabulary: 15 and 24 months, and grammar: 24 months) compared to children without DLD. This was also the case later in childhood when they were aged 8–9 years. Children with DLD had lower levels of receptive, expressive, and pragmatic language as well as lower levels of nonword repetition compared to children without DLD. As a group, the children with DLD also had significantly more emotional problems, peer problems, and conduct problems and had higher levels of hyperactivity. They were also less prosocial compared to children without DLD.

In addition to this, descriptive statistics were run to investigate earlier language profiles of children with and without DLD. In total, 63% (*n* = 209) of the children with DLD had impairment in receptive language and/or grammar at the age of 15–24 months compared to only 28% (*n* = 1,345) of children without DLD. These difficulties persisted at the age of 8–9 years when 79% (*n* = 272) of children with DLD had impairment in receptive language and/or pragmatic language compared to only 17% (*n* = 864) of children without DLD. This supports the representativeness of this community-based sample of children with DLD.

### Genetic Data

#### Quality Control

Genetic data were obtained in a preprocessed format from the ALSPAC study team and included only SNP data for requested candidate genes. Participants were genotyped using the Illumina HumanHap550 quad chip genotyping platforms by 23andme subcontracting the Wellcome Trust Sanger Institute, Cambridge, United Kingdom, and the Laboratory Corporation of America, Burlington, NC. Quality control of the data was performed prior to access. In short, individuals were excluded on the basis of gender mismatches, minimal or excessive heterozygosity, disproportionate levels of individual missingness (> 3%), and insufficient sample replication (identity by descent < 0.8). Population stratification was assessed by multidimensional scaling analysis and compared with Hapmap II (Release 22) European descent (CEU), Han Chinese, Japanese, and Yoruba reference populations; all individuals with non-European ancestry were removed. SNPs with a minor allele frequency of < 1%, a call rate of < 95%, or evidence for violations of Hardy–Weinberg equilibrium (*p* < 5E-7) were removed. Cryptic relatedness was measured as proportion of identity by descent (> 0.1). Related participants who passed all other quality control thresholds were retained during subsequent phasing and imputation. In total, 500,527 SNPs passed these quality control filters. This formed the pool for the selection of SNPs for this study.

#### Selection of SNPs

In the absence of large genome-wide studies, we sought to increase the power of our polygenic approach through the a priori selection of candidate genes after a literature-based search. Genes that had shown previous robust evidence for association to language and/or reading within the ALSPAC population using common SNPs were included in the analyses (Newbury et al., 2009; Scerri et al., 2011). Although other genes have been previously associated with language, these involved particular populations or single studies. The genes of interest and corresponding locations (hg38) were *ATP2C2* (chr16:84368615-84463732), *CMIP* (chr16:81445241-81709799), *CNTNAP2* (chr7:146116876-148415616), *DCDC2* (chr6:24174729-24357750), *FOXP2* (chr7:114426511-114693772), and *KIAA0319* (chr6:24541241-24645764). In total, there were 1,229 SNPs available at these locations. A summary of the number of SNPs by gene and location is shown in Table A2 in the Appendix.

#### Splitting of Data Set

The data set was randomly split to generate two independent data sets for genetic analyses (referred to as the discovery and replication samples) using the “generate random” command in Stata/SE 14.2. This generated two approximately equal-sized groups: discovery sample (*n* = 2,718) and replication sample (*n* = 2,717). There were no significant differences between these two sets of children on all measures of language and psychosocial outcomes (*p* > .05).

### Statistical Analyses

#### Association Analysis

SNP data were analyzed for allelic association within PLINK (Purcell et al., 2007) using linear regression models for the eight measures of language development. Eight measures were analyzed across 1,229 SNPs. The association metrics for each SNP were then used to generate a best-fit polygenic profile for each of the dependent variables (language measures) in the discovery cohort. The sensitivity of these profiles was evaluated by their ability to predict the same dependent variable within an independent replication set. This process is described in more detail below.

#### Polygenic Analysis

Polygenic profile scores were calculated in the discovery cohort within the PRSice (v1.25) package (Euesden, Lewis, & O'Reilly, 2015). This package uses the effect sizes of individual SNPs (odds ratio or β) to estimate a weighted summation score that represents all variant effects within a single measure. Polygenic profiles were evaluated across a range of *p*-value thresholds for the “base phenotype” in the discovery cohort. Best-fit scores at a given threshold were then used to predict the same base phenotype or an alternative “target” phenotype in an independent replication cohort. Polygenic profiles that significantly predict the outcome of the base measure in the replication set can be considered sensitive predictors of genetic risk. The overlaps between polygenic profiles and alternative target phenotypes can be considered to indicate the level of overlaps in genetic risk between base and target traits.

Polygenic profile scores were calculated at increments of .01 between *p*-value thresholds of 0 and .5 using the association results from the eight language measures in the discovery cohort. SNPs were thinned according to linkage disequilibrium and *p* value within the discovery data set using the “–clump” command in PRSice.

Each polygenic profile was tested within a stepwise procedure. First, the sensitivity of each of the generated polygenic profile was assessed by measuring the correlation of the score with the base language trait in the replication cohort. Second, the direct effects of the candidate genes were tested by exploring the ability of the polygenic profiles (independent variable) to predict language (DLD status) and psychosocial (SDQ subscales at the age of 11 years) outcomes as dependent variables in the replication cohort. Nominal *p* values are presented in the text with Benjamini–Hochberg adjusted values also given in Table 3.

## Results

### Associations Between Phenotypes

Pairwise correlations were run between all language and psychosocial measures at all time points (see Table 1). Overall, there were positive correlations between language measures at similar time points. For all parent report language measures that were taken when the children were 15–24 months old, there were significant correlations with medium to large effect sizes. Similarly, there were significant correlations between direct measures of expressive and receptive language as well as nonword repetition taken when the children were 8 years old. The effect sizes for these were moderate. Pragmatic language when the children were 9 years old was also positively correlated with direct measures of language when the children were 8 years old, although the effect sizes were small. Analyses of language measures across different time points showed positive correlations, but the effect sizes were generally small. Therefore, there was considerable variability between language development in early and middle childhood.

**Table 1.** Pairwise correlations between all phenotypes.

Measure, child age	1.	2.	3.	4.	5.	6.	7.	8.	9.	10.	11.	12.	13.
1. Vocabulary, 15 months	1												
2. Vocabulary, 24 months	.59***	1											
3. Receptive, 15 months	.61***	.41***	1										
4. Grammar, 24 months	.47***	.74***	.28***	1									
5. Receptive language, 8 years	.04*	.11***	.00	.09***	1								
6. Expressive language, 8 years	.10***	.21***	.03*	.19***	.37***	1							
7. Nonword repetition, 8 years	.13***	.30***	.06***	.26***	.20***	.30***	1						
8. Pragmatic language, 9 years	.08***	.17***	.05***	.14***	.10***	.15***	.16***	1					
9. Emotional problems, 11 years	.02	.01	.00	.01	−.06**	−.03*	−.02	−.22***	1				
10. Peer problems, 11 years	−.05**	−.10***	−.07***	−.07***	.01	.00	−.04**	−.30***	.35***	1			
11. Conduct problems, 11 years	−.01	−.04*	−.03	−.02	−.06***	−.08***	−.07***	−.30***	.29***	.25***	1		
12. Hyperactivity, 11 years	−.11***	−.15***	−.07***	−.12***	−.08***	−.12***	−.11***	−.44***	.25***	.23***	.47***	1	
13. Prosociality, 11 years	.09***	.07***	.13***	.05***	−.02	−.02	−.03*	.16***	−.10***	−.20***	−.41***	−.32***	1

* *p* < .05.

** *p* < .01.

*** *p* < .001.

The findings of correlations between language and psychosocial measures were mixed. Higher language ability in early childhood was associated with fewer peer problems, lower levels of hyperactivity, and higher levels of prosociality at 11 years old. On the other hand, language in early childhood was not associated with emotional and conduct problems when the children were 11 years old. Higher language ability in middle childhood was associated with fewer emotional (except for nonword repetition) and conduct problems as well as lower levels of hyperactivity. Therefore, we found some evidence for associations between language in early and middle childhood and psychosocial outcomes in middle childhood.

### Genetic Associations

Polygenic profile scores, which incorporated the effects of common variants across six language-associated genes (*ATP2C2, CMIP, CNTNAP2, DCDC2, FOXP2,* and *KIAA0319*), were considered in relation to eight language development measures, DLD status, and five SDQ subscales.

*SNP-based approach*. Although association in the ALSPAC cohort has previously been described for some SNPs included in these analyses (Newbury et al., 2011; Scerri et al., 2011), the measures and sample set used in this study differ. We therefore first evaluated associations between individual SNPs and the eight language measures to provide a baseline of association prior to polygenic investigation. As shown in Table 2, at the single SNP level, no significant association was observed for any language measure tested in the discovery cohort after correction for multiple testing. Across all 9,832 tests performed (Hauser, Moutoussis, Dayan, & Dolan, 2017), 15 had nominal *p* values at the 10^−4^ level and the minimum nominal *p* value was 1.77 × 10^−4^. This was observed between the *CNTNAP2* variant rs9648690 and receptive language at 8 years of age (all SNPs with a *p* value of 10^−4^ are shown in Table A3). In general, the language measures at 8 and 9 years of age showed a higher level of association than measures taken at 15 or 24 months of age. These data illustrate the difficulties associated with single SNP analyses across candidate genes and support the rationale for polygenic analyses, which collapse multiple genetic measures into a single factor and may be more robust to differences between sample sets.

**Table 2.** Genetic association to language measures in the discovery cohort.

Gene	Vocabulary, 15 months	Vocabulary, 24 months	Receptive language, 15 months	Grammar, 24 months	Receptive language, 8 years	Expressive language, 8 years	Nonword repetition, 8 years	Pragmatic language, 9 years	min *p*	min *p* trait
***ATP2C2***	.01513	.00543	.003633	.01061	**.000631**	**.000662**	.01517	.016	**.000631**	**Receptive language**
***CMIP***	.009171	.01004	.001037	.05581	**.00034**	**.000582**	.006129	.01474	**.00034**	**Receptive language**
***CNTNAP2***	.003648	**.000648**	.002706	.002598	**.000177**	.004907	**.000244**	**.000922**	**.000177**	**Receptive language**
***DCDC2***	.05209	.004193	.01358	.04792	**.000295**	.008146	.01634	.03848	**.000295**	**Receptive language**
***FOXP2***	.09811	.03679	.03812	.1279	.06864	.2249	.2641	.001574	.001574	Pragmatic language
***KIAA0319***	.01414	.01301	.04021	.00289	.00674	.001015	.004646	**.00076**	**.00076**	Pragmatic language
**Min *p***	.003648	**.000648**	.001037	.002598	**.000177**	**.000582**	**.000244**	**.00076**	**.000177**	**Receptive language**
**Min *p* gene**	*CNTNAP2*	*CNTNAP2*	*CMIP*	*CNTNAP2*	*CNTNAP2*	*CMIP*	*CNTNAP2*	*KIAA0319*	*CNTNAP2*	

*Note.* Single-nucleotide polymorphisms (SNPs) with a nominal *p* value of less than 9.9 × 10^−4^ are highlighted in bold. Minimum *p* values are given for all traits and genes. No single SNP was significantly associated after multiple testing corrections.

**Figure 1.**
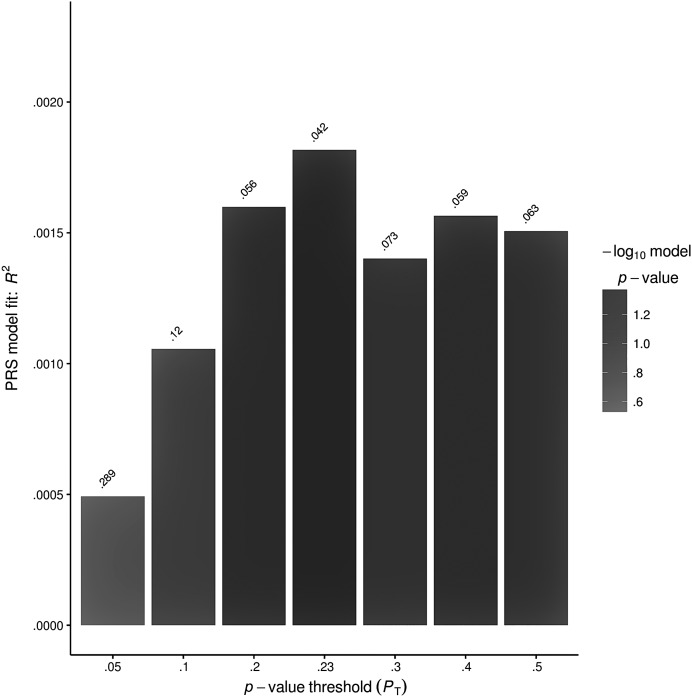
Best-fit model of genetic effects upon expressive language at 8 years of age. The best fit was found at a *p* threshold of .23 and explains .18% of trait variance (*R* ^2^) in the replication sample (*p* = .042).

*Consistency of polygenic profile scores*. Polygenic profile scores were calculated for each of the eight language measures on the basis of the effect sizes for all SNPs investigated in the discovery cohort. These scores were then evaluated for their association with the same base measure in the independent replication sample under the same threshold allowing the identification of scores and measures that provide a sensitive marker of genetic effects across samples (see Table 3). One of the eight polygenic profile scores generated (expressive language at 8 years of age) did provide a consistent marker for genetic effects upon language outcomes. The best fit for this polygenic profile score was found at a *p* threshold of .23 and explains .18% of trait variance (*R* ^2^) in the replication set (nominal *p* = .042; see Figure 1). After clumping for linkage disequilibrium and *p* value, this score was based on 65 SNPs spread across all six candidate genes included. Seven of the SNPs fell in reading candidate genes (*DCDC2* and *KIAA0319*), with the remainder falling in language candidate genes (*ATP2C2, CMIP, CNTNAP2,* and *FOXP2*).

**Table 3.** Polygenic prediction of measures and outcomes in the replication cohort.

Consistency of polygenic profile score (base trait same as target trait)						
Trait modeled		Best-fit threshold *p* value	No. SNPs in best-fit model	Proportion of trait variance explained by polygenic score (*R* ^2^)	Nominal *p* value^a^	Benjamini–Hochberg adjusted *p* value^b^
Vocabulary, 15 months		.09	32	.106%	.125	.432
Vocabulary, 24 months		.03	13	.023%	.480	.549
Receptive language, 15 months		.50	111	.034%	.385	.513
Grammar, 24 months		.16	47	.089%	.162	.432
Receptive language, 8 years		.03	19	.061%	.239	.478
Expressive language, 8 years		**.23**	**65**	**.182%**	**.042**	.336
Nonword repetition, 8 years		.09	42	.008%	.666	.666
Pragmatic language, 9 years		.01	9	.056%	.300	.480
**Overlaps in genetic effects (ability of expressive language polygenic profile to predict language and psychosocial outcomes at the age of 11 years)**						
**Base trait**	**Target trait**	**Best-fit threshold *p* value**	**No. SNPs in best-fit model**	**Proportion of trait variance explained by polygenic score (*R*^2^)**	**Nominal *p* value** ^**a**^	**Benjamini–Hochberg adjusted *p* value** ^**b**^
Expressive language, 8 years	DLD status	.08	30	.028%	.419	.628
Expressive language, 8 years	Emotional problems, 11 years	.50	123	.085%	.210	.420
Expressive language, 8 years	Peer problems, 11 years	**.06**	**27**	**.428%**	**.006**	**.036**
Expressive language, 8 years	Conduct problems, 11 years	.01	9	.094%	.189	.420
Expressive language, 8 years	Hyperactivity, 11 years	.40	97	.015%	.601	.666
Expressive language, 8 years	Prosociality, 11 years	.02	17	.010%	.666	.666

*Note.* SNPs = single-nucleotide polymorphisms; DLD = developmental language disorder.

a Models with nominal *p* values less than .05 are highlighted in bold.

b Bold results reached a significant level of association following a Benjamini–Hochberg adjustment at a false discovery rate of .05.

**Figure 2.**
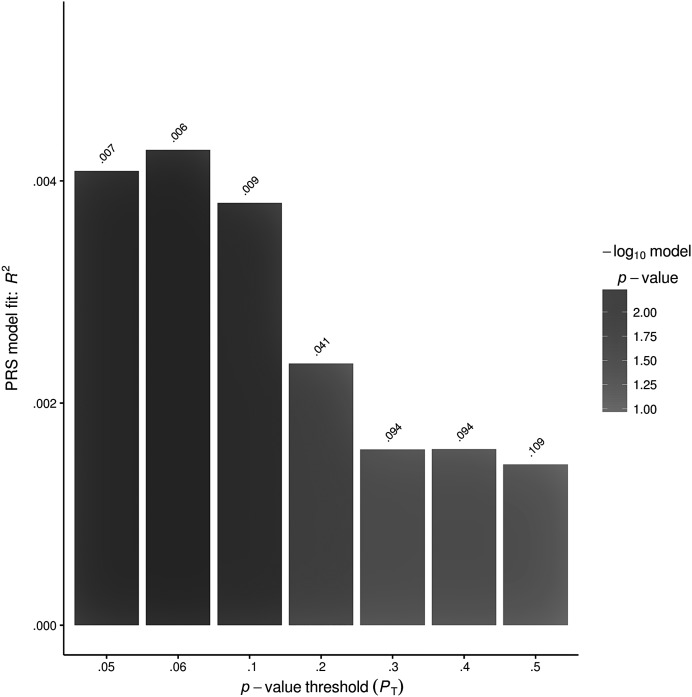
Best-fit model of genetic overlaps between expressive language at 8 years of age and peer problems. The best fit was found at a *p* threshold of .06 and explains .43% of variance (*R* ^2^) in the replication sample (*p* = .006).

*Polygenic effects on other outcomes*. The effects of the polygenic profile score upon expressive language at 8 years of age were found to be correlated with peer problems at 11 years of age suggesting genetic overlaps between expressive language and this outcome (see Table 3). To inform our understanding of the extent of overlaps, we calculated the best-fit threshold across both outcome measures in the replication set. At a *p*-value threshold of .23 (as maximized in the discovery sample), the expressive language profile score explained .22% of variation in peer problems at the age of 11 years (nominal *p* = .049). In contrast, the best fit for the prediction of peer problems at 11 years old was found at a *p* threshold of .06 and included 27 SNPs representing a subset of those contributing to expressive language. This score explained .43% of the trait variance in peer problems (*R* ^2^) in the replication set (nominal *p* = .0058, Benjamini–Hochberg corrected *p* value of .036 at a false discovery rate of .05; see Table 3 and Figure 2).

## Discussion

Using polygenic profile scores, we investigated genetic effects on language and psychosocial outcomes. Polygenic profile scores indicated some evidence of association across the SNPs tested, and the profile score for expressive language at 8 years old provided a consistent marker across the candidate genes. Of particular interest, the expressive language profile score at 8 years was significantly associated with peer problems at the age of 11 years. These findings are consistent with behavioral models in which early language difficulties increase the risk of psychosocial difficulties.

The polygenic profile scores used here were based on six genes that have previously been associated with language and/or reading. We hypothesized that this targeted approach would increase the likelihood of constructing meaningful polygenic profiles within the moderate sample sizes available (Dudbridge, 2013). In support of this hypothesis, we found that variation across these candidate genes could provide a consistent marker of the genetic effects upon expressive language, although the proportion of variance explained remained low throughout (< .3%). All genes contributed to the risk model with 5% (65 of the 1,229 SNPs tested) of variants contributing to the best-fit polygenic profile. While the proportion of variance explained is low, this is not uncommon. In other fields, such as education, earlier studies of polygenic effects explained approximately 2% of variance (Rietveld et al., 2013). In the most recent work, larger data sets have allowed for the identification of more SNPs, and so increasing the variance explained to approximately 13% (Lee et al., 2018). Similarly, larger sample sets and GWAS will allow the relative evaluation of the targeted loci studied in this article. A priori candidate genes have been substantiated within larger polygenic studies (Ritchie et al., 2019), although many candidate genes do not replicate at the GWAS level (Border et al., 2019; Johnson et al., 2017). Despite the small proportion of variance explained, our findings support the role of common variants of small effect sizes within a complex genetic model of language development and reiterate the utility of polygenic profiles, which capture multiple effects within a single score.

Furthermore, polygenic profile scores of expressive language were able to predict peer problems at the age of 11 years indicating the genetic overlaps between this measure and psychosocial outcomes (see Table 3). These findings support the previous behavioral findings, which show that language ability is correlated with psychosocial outcomes (Conti-Ramsden & Botting, 2004; Forrest et al., 2018; Mok et al., 2014). In addition, our study extends these observations in that it suggests that expressive language difficulties may increase the concomitant peer problems through shared biological pathways informing our understanding of the routes to psychosocial difficulties in children with language disorder. If replicated, such a finding is important as it may indicate the need for concurrent intervention targeting both linguistic and psychosocial development, rather than assuming early language interventions will stave off later emergence of peer problems.

We found that the best-fit *p*-value thresholds for the prediction of peer problems (*p*_T_ = .06) formed a subset of those that provided the most consistent score for expressive language alone (*p*_T_ = .23). This finding indicates that, within the polygenic model, different subsets of SNPs may be more relevant to different outcomes. Although the actual number of variants differed between thresholds, the weighting of individual SNPs was fixed between thresholds and the SNPs that contributed to peer problems formed a direct subset of those associated with expressive language. Specifically, we found that 42% (27/65) of the variants contributing to expressive language also contributed to peer problems at the age of 11 years. The relative numbers of variants from each gene were consistent across thresholds, and in general, these ratios reflected gene size. This therefore supports the relative role of all six candidate genes in language development.

The methods reported here provide a useful approach to the investigation of relationships between genetic risks and environmental factors. Through the application of polygenic profiles, we have demonstrated potential shared genetic relationships between expressive language and peer problems. These findings provide further evidence of the role of genes in language development and emphasize the importance of larger-scale studies to identify specific factors that moderate risk and mediate positive psychosocial outcomes for children affected by language disorder. Likewise, investigations of genetic comorbidities between language disorder and psychosocial outcomes represent an interesting future direction (Bulik-Sullivan et al., 2015). Application of this approach has revealed substantial overlaps between neurodevelopmental disorders and in relation to educational attainment (Gialluisi et al., 2019; Grove et al., 2019). Such studies allow biological insights into disease mechanisms and may help to target intervention to individuals who will best benefit from additional support in terms of long-term outcomes.

An additional finding of note concerns the prosocial subscale of the SDQ. Previous evidence indicates that individuals with DLD show relatively stable prosociality scores from late childhood to 24 years old (Lindsay & Dockrell, 2012; Toseeb et al., 2017). The finding that prosociality was within the normal range for both those with DLD and those without (see Table A1) provides further support for the inference that prosociality is a characteristic of relative strength in young people with DLD (in contrast to their less favorable scores on the other SDQ subscales). We obtained no indication that prosociality scores could be predicted by polygenic profiles, suggesting that this aspect of psychosocial functioning may either be influenced by other genetic factors or be nurtured by socialization processes (or some interaction of these factors). We cannot resolve this issue on the basis of the present data, but the findings do add to an accumulating body of evidence that this strength in prosociality in those with DLD may be sufficiently robust to warrant incorporation in therapeutic work (Conti-Ramsden & Durkin, 2016; Toseeb et al., 2017).

When considering the findings of this study, some drawbacks should be borne in mind. This study considers only a small number of genes that, between them, account for only a small proportion of genetic risk. Thus, although the patterns that we observed support existing behavioral data, we must qualify these observations with the fact that these effects only represent a small proportion of genetic liability and a small part of the complex picture of risks relevant to language disorders. Nonetheless, in the absence of a large-scale picture of genome-wide effects in language disorder, the focus of this work upon a small set of robust candidate genes allowed the derivation of consistent polygenic profile of language disorder, albeit with a small effect size. A functional candidate approach, such as the one we have reported here, has previously been shown to provide an accurate way of focusing polygenic profile studies, for example, in fibrogen pathways in cardiovascular disease (Cronjé et al., 2017). In this instance, the use of a specified set of candidate genes allowed the further exploration of relative subsets in relation to outcome measures. This study illustrates the utility of polygenic models in the study of language disorders and, when larger samples become available, can be extended to a genome-wide model.

We should also note that only one of the *p* values found in the current study survived multiple testing corrections. Although polygenic profiles allow the reduction of dimensions through the consideration of a single weighted risk score, they still involve multiple tests, especially when high-resolution best-fit modeling is employed (Euesden et al., 2015). The use of hypothesis-driven, predefined models offsets this issue to some extent as we have some a priori expectation of the patterns we will observe from the behavioral literature. Nonetheless, these findings require extension to, and replication in, larger, independent data sets to claim significance.

Overall, our study illustrates the utility of polygenic methods in the study of children's language development. We found preliminary evidence that polygenic profiles for expressive language can be used to predict expressive language and peer problems in an independent sample. Our findings point to particular language and psychosocial outcomes that appear to be associated with genetic risk of language disorder.

## Figures and Tables

**Table A3.**
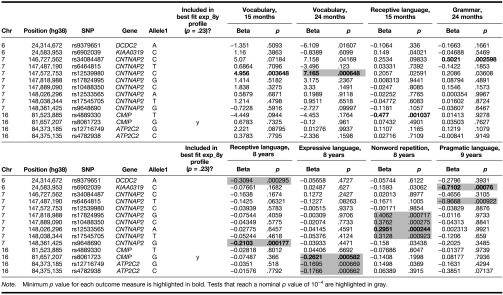
Single-nucleotide polymorphisms (SNPs) in analyses that reached nominal level of significance (*p* = 10^−4^)

## Appendix

**Table A1.** Group level comparisons for all variables of interest.

Measure, child age	Overall			Without DLD	With DLD	Mean difference [95% CI]	Test statistics	Effect size
	*n*	Range	*M* (*SD*)	*M* (*SD*)	*M* (*SD*)			
**Language and communication measures**								
Vocabulary, 15 months	5,259	0–268	88.36 (43.23)	89.37 (43.32)	73.43 (39.03)	15.94 [11.18, 20.71]	*t*(5,257) = 6.56***	0.37
Vocabulary, 24 months	5,205	0–246	158.66 (53.16)	161.57 (51.88)	116.11 (53.34)	45.46 [39.69, 51.23]	*t*(5,203) = 15.44***	0.87
Receptive, 15 months	5,263	0–12	9.18 (2.41)	9.20 (2.40)	8.90 (2.50)	0.30 [0.03, 0.57]	*t*(5,261) = 2.20*	0.12
Grammar, 24 months	5,205	0–8	3.62 (2.55)	3.74 (2.53)	1.84 (2.05)	1.90 [1.63, 2.18]	*t*(5,203) = 13.42***	0.76
Receptive language, 8 years	5,429	2–15	7.61 (1.87)	7.73 (1.80)	5.86 (1.99)	1.87 [1.68, 2.07]	*t*(5,427) = 18.62***	1.03
Expressive language, 8 years	5,416	0–10	7.65 (1.68)	7.74 (1.61)	6.26 (2.04)	1.48 [1.30, 1.66]	*t*(5,414) = 16.22***	0.90
Nonword repetition, 8 years	5,424	0–12	7.41 (2.42)	7.64 (2.27)	4.11 (2.13)	3.53 [3.28, 3.78]	*t*(5,422) = 28.03***	1.56
Pragmatic language, 9 years	4,464	98–162	151.83 (6.62)	152.43 (5.87)	144.12 (10.03)	8.32 [7.61, 9.03]	*t*(4,462) = 22.94***	1.33
**Psychosocial outcomes**								
Emotional problems, 11 years	4,308	0–10	1.36 (1.64)	1.34 (1.64)	1.65 (1.744)	−0.31 [−0.51, −0.11]	*t*(4306) = −3.05**	−0.19
Peer problems, 11 years	4,157	0–9	0.95 (1.39)	0.92 (1.36)	1.47 (1.70)	−0.55 [−0.72, −0.38]	*t*(4155) = −6.37***	−0.40
Conduct problems, 11 years	4,290	0–9	1.11 (1.33)	1.08 (1.31)	1.56 (1.56)	−0.49 [−0.65, −0.33]	*t*(4288) = −6.02***	−0.37
Hyperactivity, 11 years	4,283	0–10	2.56 (2.13)	2.48 (2.08)	3.76 (2.50)	−1.28 [−1.54, −1.03]	*t*(4281) = −9.83***	−0.61
Prosociality, 11 years	4,305	0–10	8.40 (1.62)	8.41 (1.62)	8.20 (1.69)	0.22 [0.02, 0.41]	*t*(4303) = 2.18*	0.13

*Note.* DLD = developmental language disorder; CI = confidence interval.

* *p* < .05.

** *p* < .01.

*** *p* < .001.

**Table A2.** Summary of single-nucleotide polymorphisms (SNPs) by gene.

Gene	Number of SNPs in analysis	ALSPAC coordinates (hg38)
*CMIP*	172	chr16:81445241-81709799
*ATP2C2*	158	chr16:84368615-84463732
*DCDC2*	100	chr6:24174729-24357750
*KIAA0319*	52	chr6:24541241-24645764
*FOXP2*	27	chr7:114426511-114693772
*CNTNAP2*	720	chr7:146116876-148415616

*Note.* ALSPAC = Avon Longitudinal Study of Parents and Children.
